# Man and Animals

**Published:** 1882-12

**Authors:** 


### MAN AND ANIMALS.

There can be no doubt that dogs associate with barking in certain tones special emotional states in their companions. In fact, it is probable that dogs can in this way communicate with each other a wide range of states of feeling. But these states are present states, not states past or future. They are their own states, not the states of others. A dog can call his companion's attention to a worriable cat, or he may have his attention roused by my exclaiming "cat." But no dog could tell his companion of the successful "worry" he had just enjoyed, or suggest that they should go out for a "worry" to-morrow morning. And here we come upon what seems to me the fact which raises man so immeasurably above the level of the brute. The brute has to be contented with the experience he inherits or individually acquires. Man, through language, spoken or written, profits by the experience of his fellows. Even the most savage tribe has traditions extending back to the father's father (Sproat). And the civilized man—has he not in his libraries the recorded results of many centuries of ever-widening experience and ever-deepening thought? Thus it is that language has made us men. By means of language, and language alone, has human thought become possible. This it is which has placed so enormous a gap between the mind of man and the mind of the dog. Through language each human being becomes the inheritor of the accumulated thought and experience of the whole human race. Through language has the higher abstract thought become possible.—*London Nature.*

A YOUNG NOBLEMAN in a frightful railway accident missed his valet. One of the guards came up to him and said, "My lord, we have found your servant, but he is cut in two." "Aw, is he?" said the young man, with a Dundreary drawl, but with a trace of anxiety depicted on his countenance. "Will you be good enough to see in which half he has got the key of my carpet-bag?"

